# Making sense of invisible densities in single-particle cryo-EM

**DOI:** 10.1107/S2052252526002952

**Published:** 2026-04-24

**Authors:** Xiaoxuan Lin, Yifan Cheng

**Affiliations:** a Howard Hughes Medical Institute University of California San Francisco San Francisco California USA; b Department of Biochemistry and Biophysics University of California San Francisco San Francisco California USA; University of Michigan, USA

**Keywords:** cryo-EM, protein structures, protein dynamics, hydrogen–deuterium exchange mass spectrometry

## Abstract

In the era of single-particle cryogenic electron microscopy (cryo-EM) and AI-driven protein structure prediction, obtaining high-resolution protein structures, either experimentally or computationally, has become increasingly routine. Yet studying and understanding protein dynamics remains challenging. In single-particle cryo-EM, protein dynamics are most obviously manifested as poor local resolution or disappearing densities in specific regions of a reconstruction. No method is yet available to computationally generate conformational ensembles that fully deconvolute these experimental observations. When dynamics are key to understanding protein function, it is clear to us that introducing new experimental approaches is necessary to close this gap and make sense of invisible densities in single-particle cryo-EM.

## Abstract 2

Protein dynamics are often manifested as disappearing densities in cryo-EM structures. Making sense of such disappearing densities in order to understand their biological implications requires approaches beyond high-resolution structures.

## 1. Introduction

Richard Feynman famously advocated the ‘just look at the thing’ approach as an intuitive way to understand biological systems (Feynman, 1960 ▸). This advice has perhaps never been more literal and pertinent than in the era of single-particle cryo-EM. Over the past decade, advances in direct electron detectors and image processing have made high-resolution structure determination routine for biological studies. The ability to visualize macromolecules at near-atomic detail has transformed, with remarkable speed, how we think about protein function (Cheng, 2018 ▸; Nogales, 2016 ▸). Drawn to this power of ‘seeing is believing’, our laboratory has been devoted to advancing cryo-EM methodology and applying it across a wide range of biological problems. These experiences have brought us to a more fundamental question: what are the limits of simply ‘looking’ at a structure, and what do we do when that is no longer sufficient to tell us how a protein works?

## 2. The conundrum of invisibility

A major obstacle in achieving high-resolution structure determination by single-particle cryo-EM is sample heterogeneity, particularly that arising from conformational dynamics. Common strategies to address this challenge, with the goal of obtaining high-resolution reconstructions, include biochemical stabilization of the target protein and computational isolation of homogeneous subsets. In parallel, various computational algorithms have been developed to model continuous conformational changes and generate molecular movies describing protein dynamics (Choi *et al.*, 2025 ▸; Nakane & Scheres, 2021 ▸; Punjani & Fleet, 2021 ▸; Zhong *et al.*, 2021 ▸). Despite applying these strategies in our own studies, we repeatedly encounter a conundrum, which is also familiar to the broader structural biology community: while parts of a protein are well resolved to high resolution, other parts of the same protein consistently fail to produce interpretable density, no matter how the data are processed (Campbell *et al.*, 2020 ▸; Jin *et al.*, 2024 ▸; Zimanyi *et al.*, 2025 ▸). In such cases, all available image processing methods, including extensive particle classification, heterogeneous refinement, or 3D variability analysis (3DVA), fail to recover meaningful intermediates. These unresolved regions thus become ‘dead zones’ in single-particle cryo-EM: without clearly resolved density, structure-based interpretation comes to a grinding halt. We often fall back on familiar language, describing such regions as ‘dynamic’, ‘flexible’, or ‘heterogeneous’. Yet these terms obscure more than they explain. Do the missing densities reflect large-scale domain motions, local plasticity within a folded structure, or partial unfolding? The ambiguity deepens when perturbations such as ligand binding cause density to disappear or reappear, leaving us to conclude, often unsatisfyingly, that a region has become ‘more’ or ‘less’ flexible. For the sake of resolving unresolvable densities, it is often tempting to biochemically stabilize these regions or the whole protein, as once demonstrated in efforts to crystallize G-protein-coupled receptors (Vaidehi *et al.*, 2016 ▸). But what if this ‘flexibility’ is itself a part of the protein function? Then, a rigidified structure risks becoming a visually satisfying picture but with little biological meaning. A related conundrum arises when a clear phenotype occurs without apparent conformational changes linking discrete structural and functional endpoints (Jin *et al.*, 2024 ▸). We often describe such behavior as ‘allostery’ but remain puzzled by the exact molecular and thermodynamic basis behind it.

**Figure 1:**
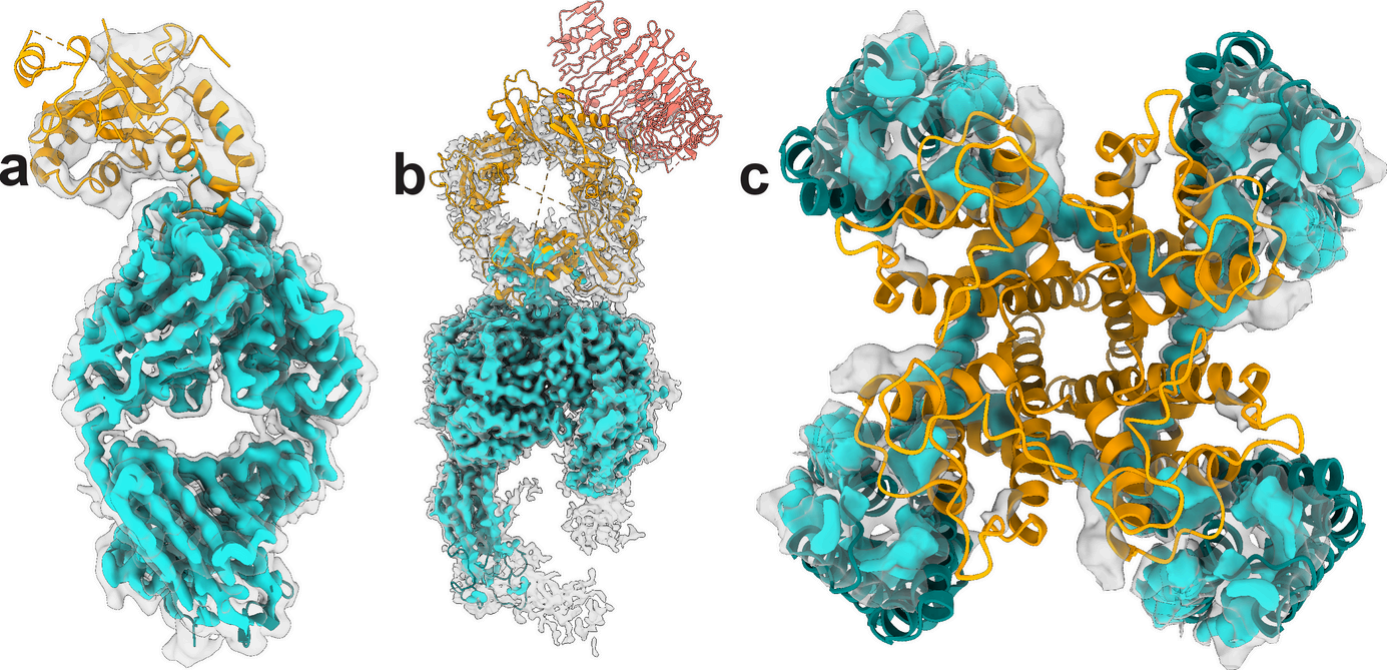
Examples of single-particle cryo-EM density maps with missing densities. (*a*) Single-particle cryo-EM map of human herpesvirus protease (orange) bound to a Fab (teal) with the separate atomic models docked. The Fab and the protease epitope are well resolved. Resolution decreases for the remaining part of the protease with densities disappearing in the distal regions. (*b*) Single-particle cryo-EM map of integrin αvβ8 (teal) bound to L-TGF-β/GARP (orange/salmon), with the atomic model of αvβ8/L-TGF-β/GARP docked. The resolution of L-TGF-β decreases from the integrin binding site to the distal region. GARP, which is covalently linked to L-TGF-β, is completely invisible in the density map. (*c*) A top view of the transmembrane domain of the TRPM8 channel from a typical reconstruction without extensive classification with the atomic model docked. The voltage-sensing-like domains (teal) are resolved, but densities for most of the pore domain (orange) are not visible. In all examples, the same density map is shown in high threshold (cyan) and low threshold (transparent gray).

These conundrums are clearly illustrated in several of our own recent studies. For example, in our analysis of human herpesvirus protease bound to an inhibitory Fab, we resolved multiple 3D classes with distinct Fab-binding poses, accompanied by a progressive loss of density extending away from the epitope into the distal active site [Fig. 1 ▸(*a*)] (Zimanyi *et al.*, 2025 ▸). This suggested that, in addition to a simple rocking motion of the protease around the bound Fab, the body of the protease must sample substantial conformational dynamics, or even partial unfolding. This raised a key question: how does antibody binding inhibit catalysis when the structural path between the epitope and the catalytic site is invisible?

A similar puzzle was presented in our study of TGF-β activation by integrin (Jin *et al.*, 2024 ▸). In the multiple conformational subclasses of the complex, density progressively disappeared from the integrin binding site toward mature TGF-β [Fig. 1 ▸(*b*)]. These observations challenged any interpretation based on discrete structural transitions and instead led us to explore a dynamic allostery model that drives TGF-β activation.

A related challenge arose in our studies of TRPM8, a menthol- and cold-activated ion channel. Early published cryo-EM reconstructions (Yin *et al.*, 2018 ▸), and our own unpublished reconstructions, consistently lacked clearly resolved density in the pore domain [Fig. 1 ▸(*c*)], precluding structural conclusions about channel gating. This impasse persisted until recently, when we combined cryo-EM with energetic measurements that revealed conformational ensembles underlying TRPM8’s cold sensitivity (Choi *et al.*, 2026 ▸).

From these examples, and many others, we have come to view biological questions not as simple structure–function relationships, but as ensemble–function relationships – one defined by structural populations, energetics, and their combined relevance to function and phenotype.

## 3. From structure to ensemble

What, then, is the nature of the ‘flexibility’ encoded in regions that escape from being resolved in cryo-EM density maps? Local resolution and temperature factor can offer qualitative hints about motion, but these descriptive phenomenological metrics fall short in providing biologically meaningful explanations. In our experience, simply labeling a region as ‘flexible’ often obscures, rather than resolves, the underlying mechanistic questions.

Any spontaneous event must ultimately be driven by free energy, through shifts in the population of all possible states that a protein samples according to the Boltzmann distribution, even when no apparent structural changes can be visualized. This realization led us to conclude that understanding protein function requires more than structural snapshots, but rather access to both conformational ensembles, represented by either discrete conformational classes or continuous protein motion captured in cryo-EM, and the underlying energetics. This un-met need pointed us in the direction of hydrogen–deuterium exchange (HDX), which reports on rare, high-energy states that can dominate functional outcomes, yet are invisible to single particle cryo-EM or X-ray crystallography.

Proteins in their native states experience transient disruptions of backbone hydrogen bonds due to spontaneous thermal fluctuations, which permit amide protons to exchange with protons in the solvent. This hydrogen exchange (HX) can be measured experimentally by nuclear magnetic resonance or mass spectrometry when deuterium, instead of hydrogen, is present in the surrounding buffer. The frequency of HX events, ranging from nearly continuous, as in unfolded domains, to extremely rare, such as one in a million for very stable folds, is controlled by protein dynamics and energetics, as first articulated by Linderstrøm-Lang in the early 1950s (Englander & Kallenbach, 1983 ▸; Hvidt & Pedersen, 1974 ▸). While cryo-EM structures capture snapshots near the bottom of a free-energy well, HX rates are determined by excursions across the entire free-energy landscape, including the short-lived and sparsely populated states. Coupling HX with mass spectrometry and proteolytic fragmentation has made it possible to access this information at the near-amino-acid level for large protein complexes (Engen & Komives, 2020 ▸; Englander, 2006 ▸; Mayne *et al.*, 2011 ▸), often in regions where cryo-EM density is absent. This complementarity makes HX a natural extension of cryo-EM.

In the following section, we illustrate this perspective through our recent work on the cold sensor TRPM8, where integrating cryo-EM with hydrogen–deuterium exchange mass spectrometry (HDX-MS) led us to connect invisible structure with energetics, structural ensembles and thermosensation.

## 4. Structural energetics – a case study

Early structural efforts on the cold and menthol sensor TRPM8 yielded an otherwise well resolved channel structure, but in which the pore domain remained persistently fuzzy [Fig. 1 ▸(*c*)], hinting at extreme dynamics that we could not interpret structurally. Only recently, by examining TRPM8 in cell membrane vesicles, could we identify a previously unseen ‘semi-swapped’ tetrameric architecture with a novel pore organization distinct from the canonical ‘fully swapped’ arrangement (Choi *et al.*, 2026 ▸). This raised a key question: is the unresolved pore domain a consequence of continuous transitions between these architectures? And if so, could such transitions be part of the cold sensing mechanism? Extensive 3D classification revealed many less well resolved, presumptive ‘intermediate’ substates. Are these snapshots of a functional pathway?

Particle counts suggested that the semi-swapped state increases upon menthol binding, but structural statistics alone left us uneasy. How many structural forms coexist and how many of them are biologically meaningful? Have the right particles been picked? Has particle selection favored one state over another? In our HDX-MS measurements, we observed two stable energy states that freely interconvert in solution, with population shifts induced by menthol. This enhanced our confidence that the fully- and semi-swapped states coexist dynamically, with the numerous 3D ‘intermediate’ classes representing the pathway connecting them.

Discovery of the ‘semi-swapped’ channel architecture may just be the tip of the iceberg. The field has been fascinated by the unusually robust temperature sensitivity of TRP channels. Structural heterogeneity is likely amplified by these channels’ thermosensitivity, generating substates too transient or numerous to register as major structural subclasses. Even when identifiable, it is difficult to determine which conformational changes drive temperature sensitivity versus those that are incidental. Integrating cryo-EM, temperature-dependent HDX-MS, mutagenesis and cellular assays now allows us to access both structural and energetic contributions from local regions, together enabling us to propose a more holistic energetic landscape of temperature-evoked TRP channel gating that neither approach alone could provide. This perspective enlightened our approach from simply asking ‘what are the structural changes upon cold’ to also understanding ‘what energetic forces drive functional transitions’.

## 5. Outlook

It is often a common practice in single-particle cryo-EM, following classifications, to discard reconstructions lacking resolution. Yet numerous of our studies suggest that these low-resolution regions can reflect functionally important dynamic movements. Protein function is frequently tuned by dynamics without discrete conformational changes, exemplified by dynamic allostery (Jin *et al.*, 2024 ▸; Campitelli *et al.*, 2025 ▸; Popovych *et al.*, 2006 ▸). We remain fascinated by single-particle cryo-EM as a way to reveal how molecules work by simply ‘looking’ at the ‘thing’. But over time, we have come to appreciate that the highest-resolution structures capture mostly long-lived, low-energy states, while functionally active states are often rarely populated and hard to resolve. Recognizing this has motivated us to expand our focus from pursuing the highest-resolution structures towards understanding the dynamic processes underlying physiologic function.

HDX-MS complements 3D structures by providing quantitative assessments to these high-energy, transient states, while offering thermodynamic information at the protein segment level. Integrating these two approaches has brought us closer to achieving a holistic view of protein behavior, as illustrated by our recent work on temperature-sensitive ion channels (Choi *et al.*, 2026 ▸). Understanding how protein dynamics drive phenotype will require us to continuously improve and expand our toolkits, from single-molecule fluorescence resonance energy transfer and molecular dynamics simulations to AI-driven protein structure prediction and protein design, and beyond. For example, one can envision using all measurable structural and thermodynamic parameters as constraints to generate conformational ensembles through AI-driven atomic structure prediction. Only by integrating complementary approaches can we hope to map the complete landscape of protein function to achieve a deeper understanding of the fundamental principles that govern biological processes.
